# Case Report: A seventy-year-stable chest wall bronchogenic cyst with sudden enlargement: coincidence or connection with a concurrent thymic carcinoma?

**DOI:** 10.3389/fmed.2026.1815680

**Published:** 2026-05-08

**Authors:** Jiawei Huang, Kairen Xie, Huajun Li, Jing Xie, Yunjie Liang, Ying Chen, Wanli Lin

**Affiliations:** 1 Department of Thoracic Surgery, Affiliated Gaozhou People’s Hospital, Guangdong Medical University, Maoming, Guangdong, China; 2 Guangdong Medical University, Zhanjiang, Guangdong, China; 3 Department of Surgery, Xincheng County People's Hospital, Xincheng, Guangxi, China

**Keywords:** bronchogenic cyst, chest wall, cyst enlargement, paracrine stimulation, thymic carcinoma

## Abstract

**Introduction:**

Bronchogenic cysts arising in the chest wall are uncommon. This report describes a unique case of a chest wall bronchogenic cyst that exhibited delayed proliferation after seventy years of stability, concurrently presenting with a separate anterior mediastinal thymic carcinoma.

**Case presentation:**

A 75-year-old woman presented with a six-month history of gradual enlargement of a long-standing, previously stable suprasternal mass. Computed tomography revealed a well-circumscribed subcutaneous cyst and a separate anterior mediastinal mass. Both lesions were completely excised. Histopathology confirmed the chest wall lesion as a bronchogenic cyst and the mediastinal mass as a poorly differentiated squamous cell carcinoma of thymic origin (Masaoka-Koga stage I). The patient recovered well with no recurrence at follow-up.

**Discussion:**

While sudden enlargement of bronchogenic cysts is documented, delayed growth after an exceptionally prolonged quiescent period is exceedingly rare. The concurrent discovery of a histologically distinct yet anatomically adjacent thymic carcinoma raises the question of a potential pathological interaction beyond coincidence. Common mechanisms for cyst enlargement, such as infection, haemorrhage, or secretory obstruction, were absent. We hypothesise that paracrine stimulation by factors secreted from the thymic carcinoma may have disrupted the cyst’s homeostasis, prompting its secondary growth. This hypothesis warrants further investigation through molecular analysis of similar cases.

**Conclusion:**

We report an exceptionally rare instance of delayed growth in a thoracic wall bronchogenic cyst, occurring after more than seven decades of quiescence and coincident with an anatomically adjacent thymic squamous cell carcinoma. This finding underscores the necessity for comprehensive evaluation of long-standing masses that undergo change; it also provides significant clinical insights, documents a unique case, and suggests avenues for investigating the underlying mechanism.

## Introduction

Bronchogenic cysts originate from embryonic foregut abnormalities and are most commonly located within the mediastinum or pulmonary parenchyma. Their occurrence in the chest wall is relatively uncommon. This report presents a case of a chest wall bronchogenic cyst that remained stable for 70 years before progressively enlarging over a six-month period. Diagnostic investigations concurrently revealed a separate anterior mediastinal mass, which was histologically confirmed as a poorly differentiated squamous cell carcinoma of thymic origin. To our knowledge, this is a rare case of a bronchogenic cyst exhibiting delayed proliferation following a prolonged period of stability, concurrently presenting with a malignant thymic tumour. This unusual association may imply a more complex relationship rather than a coincidental occurrence, warranting further consideration.

## Case

**Figure 1:**
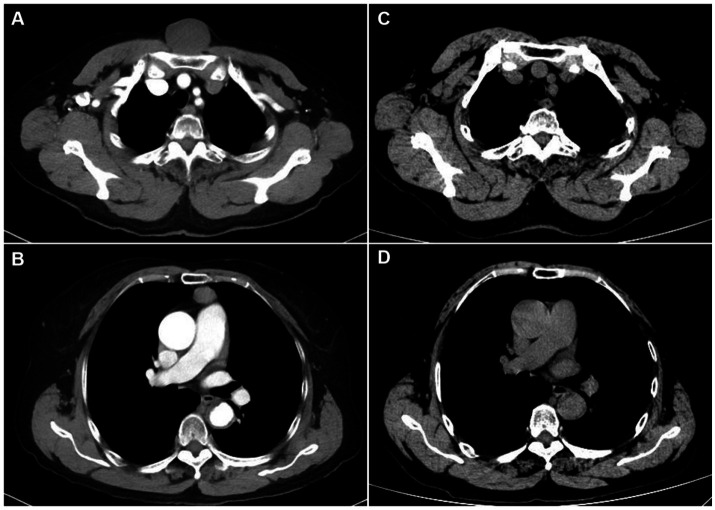
Preoperative and follow-up chest computed tomography (CT) images. **(A)** On 26 March 2023, chest CT demonstrates a well-defined, round subcutaneous lesion (approximately 44 mm × 43 mm) anterior and superior to the manubrium. **(B)** On 26 March 2023, chest CT reveals a well-defined mass (approximately 22 mm × 16 mm) in the anterior mediastinum. **(C)** On 11 October 2024, follow-up chest CT shows no evidence of tumour recurrence at the previous subcutaneous site. **(D)** On 11 October 2024, follow-up chest CT shows no evidence of tumour recurrence at the previous anterior mediastinal site.

A 75-year-old woman was evaluated on 20 March 2023 for a chest wall mass. The lesion had been identified by the patient approximately seventy years earlier and had subsequently remained stable until a noticeable enlargement occurred over the six months preceding presentation. This recent growth was gradual and asymptomatic, with no associated pain, systemic symptoms, or changes to the overlying skin. Her past medical history was otherwise unremarkable, and she was on no regular medications. On physical examination, a firm, non-fluctuant mass with a relatively fixed base was palpable in the suprasternal region. Chest computed tomography (CT) revealed a well-circumscribed, roughly circular subcutaneous lesion measuring approximately 44 mm × 43 mm anterior and superior to the manubrium (Figure 1A). Concurrently, a separate, well-defined mass measuring approximately 22 mm × 16 mm was identified within the anterior mediastinum (Figure 1B).

**Figure 2:**
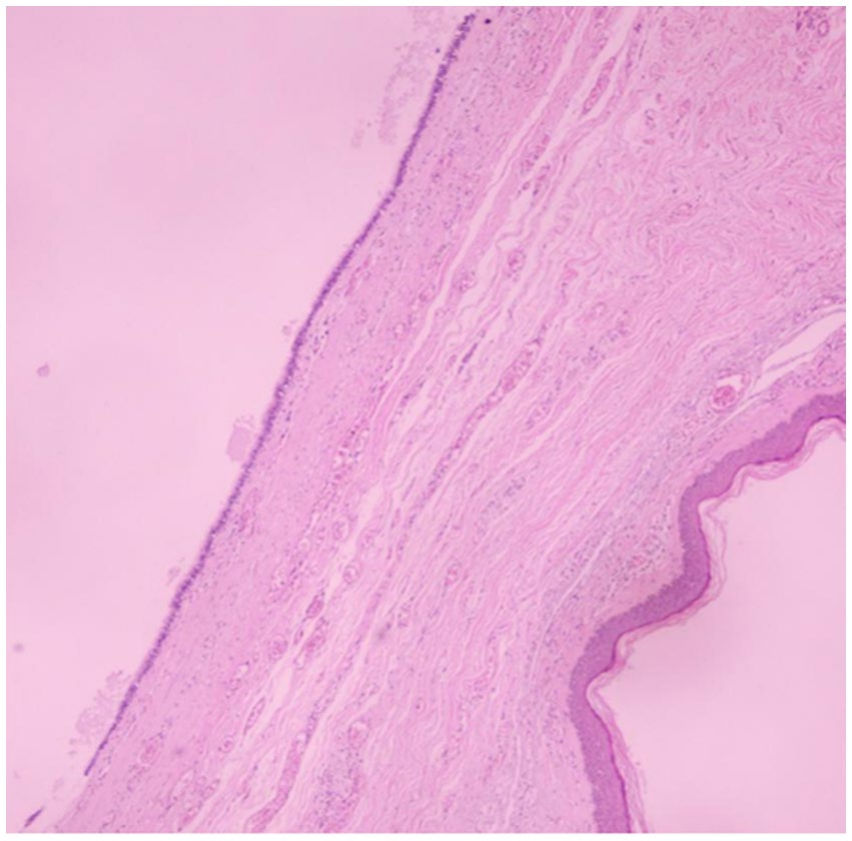
Histopathological confirmation of bronchogenic cyst. Photomicrograph confirming a bronchogenic cyst. The cyst wall is lined by characteristic pseudostratified ciliated columnar epithelium.

On 28 March 2023, the patient underwent a combined surgical procedure. The anterior mediastinal mass was resected via a subxiphoid approach under video-assisted thoracic surgery (VATS) guidance. Subsequently, the subcutaneous chest wall mass was excised through a direct cutaneous incision. Intraoperatively, both lesions were found to be well-encapsulated and clearly demarcated from the surrounding tissues. Histopathological examination confirmed the chest wall mass as a bronchogenic cyst (Figure 2) and the mediastinal mass as a poorly differentiated squamous cell carcinoma of thymic origin. Immunohistochemical analysis of the thymic carcinoma was positive for P63, CD117, and CD5, and negative for CK7, TDT, CD20, CD3, and TTF-1. The Ki-67 proliferation index was approximately 10% in hotspot areas. The postoperative staging of the thymic malignancy was Masaoka-Koga stage I. The patient recovered well after surgery. A follow-up CT scan in October 2024 showed no tumour recurrence at either site (Figures 1C,D). Telephone follow-up until December 2025 indicated the patient remained well with no specific complaints. A detailed clinical timeline is summarized in Table 1.

**Table 1:** Clinical timeline.

Time point	Clinical details
*Circa* 1950s	An anterior chest wall mass was first identified.
*Circa* 2022	The previously identified chest wall mass was noted to have increased in size compared with prior findings.
20-Mar-2023	CT demonstrated a chest wall mass measuring approximately 44 mm × 43 mm. An incidental mediastinal mass measuring approximately 22 mm × 16 mm was also detected in the anterior mediastinum.
28-Mar-2023	Surgical intervention was performed. Histopathological examination: – Chest wall lesion: bronchogenic cyst – Anterior mediastinal lesion: squamous cell carcinoma of thymic origin
11-Oct-2024	CT demonstrated no evidence of disease recurrence.
Dec-2025	Telephone follow-up was completed; no abnormal or concerning findings were reported.

## Discussion

Bronchogenic cysts usually present with non-specific clinical features and are commonly considered benign. When located in the anterior chest wall, differential diagnoses include epidermoid cysts, sebaceous cysts, and soft tissue tumours. Clinically, bronchogenic cysts have normal overlying skin, unlike epidermoid or sebaceous cysts which often show a visible skin punctum. Radiologically, CT and ultrasound can reliably distinguish bronchogenic cysts (water-density, anechoic, no enhancement) from soft tissue tumours (vascularity, enhancement). Pathology confirms the diagnosis: bronchogenic cysts are lined by pseudostratified ciliated columnar epithelium. Symptoms are usually attributable to local compression or secondary infection. Due to the potential risks of infection, compression, enlargement, and even rare malignant transformation (1, 2), complete surgical excision of the cyst wall is widely recommended. In the present case, the cyst base was free of adhesions to adjacent structures intraoperatively, facilitating uncomplicated complete excision.

Bronchogenic cysts have been reported to occasionally enlarge suddenly after a prolonged period of stability (3). In this particular case, the thoracic wall bronchogenic cyst remained stable for over 70 years, aligning with their typically quiescent or slow-growing behaviour. Nevertheless, progressive enlargement after such an exceptionally long quiescent period—termed ‘delayed growth’—is exceedingly rare in the published literature. Of particular note, CT imaging unexpectedly identified an adjacent anterior mediastinal thymic mass. The question of whether this finding is merely coincidental or indicative of a more intricate pathological interaction is of paramount importance. This ambiguity warrants further consideration.

Our literature review summarises several potential pathophysiological mechanisms for the sudden enlargement of bronchogenic cysts after prolonged stability: (1) increased secretion of cystic fluid or obstructed drainage, leading to elevated intra-cystic pressure; (2) acute dilatation due to secondary intracystic infection or haemorrhage; and (3) altered proliferative activity of the cyst wall’s epithelial or stromal cells, such as that seen in malignant transformation (2). In our case, preoperative assessment revealed no clinical signs of infection (e.g., erythema, warmth, or pain). Intraoperatively, the cyst capsule was intact with no adhesions to surrounding tissues. Postoperatively, histopathological examination confirmed the absence of infection, haemorrhage, or significant inflammatory infiltration. Therefore, the observed phenomenon of delayed rapid growth cannot be adequately explained by mechanisms of secretory accumulation and pressure elevation alone.

In this case, the two masses are of distinct tissue origin: the bronchogenic cyst derives from the foregut, whereas the thymic carcinoma originates from the thymic epithelium. It is well documented that thymic carcinoma cells can secrete a variety of growth factors [e.g., vascular endothelial growth factor (VEGF) and fibroblast growth factor (FGF)] and cytokines, and can also mediate local inflammatory responses (4, 5).

One possible explanation is that such secreted factors may diffuse into the adjacent bronchogenic cyst wall, potentially influencing the proliferation, secretory function, or general metabolic activity of its resident epithelial and stromal cells. Such paracrine stimulation might, in theory, disrupt the cyst’s long-maintained homeostatic balance, potentially contributing to its secondary enlargement. While this paracrine hypothesis remains speculative and requires further validation, it raises a key question: could the adjacent thymic carcinoma have acted as an ‘initiating factor’ by releasing bioactive substances that may have triggered the delayed growth of the bronchogenic cyst?

Future studies should include systematic molecular analysis of cyst wall tissue from similar cases—including assessments of growth factor receptor expression and proliferation marker activity—with comparisons to isolated, stable bronchogenic cysts. Additionally, analysis of cytokine profile within the cyst fluid or the pericystic microenvironment could provide valuable insights into the local inflammatory state and help elucidate the underlying mechanisms. This report documents a rare clinical observation and offers preliminary pathogenic considerations. We emphasize the exploratory nature of these hypotheses and acknowledge alternative explanations, with the goal of prompting further discussion and investigation.

## Conclusion

We report an exceptionally rare instance of delayed growth in a thoracic wall bronchogenic cyst, occurring after more than seven decades of quiescence and coincident with an anatomically adjacent thymic squamous cell carcinoma. This finding underscores the necessity for comprehensive evaluation of long-standing masses that undergo change; it also provides significant clinical insights, documents a unique case, and suggests avenues for investigating the underlying mechanism.

## Data Availability

The original contributions presented in the study are included in the article/supplementary material, further inquiries can be directed to the corresponding author/s.
